# Radiographic evaluation of narrow-diameter implants from different systems for two years

**DOI:** 10.3389/fbioe.2025.1631745

**Published:** 2025-09-01

**Authors:** Xudong Fan, Xiaoya Cheng, Yuanyuan Wang

**Affiliations:** ^1^ Department of Stomatology, Wuhan No.1 Hospital (Wuhan Hospital of Traditional Chinese and Western Medicine), Wuhan, Hubei, China; ^2^ Department of Zhao Jiatiao Outpatient Center, School and Hospital of Stomatology, Wuhan University, Wuhan, Hubei, China

**Keywords:** narrow-diameter implants, marginal bone loss, implant survival, radiographic evaluation, titanium-zirconium alloy, peri-implant bone level

## Abstract

**Background:**

Narrow-diameter implants (NDIs) offer a viable alternative to bone augmentation in sites with limited alveolar ridge width. This study aimed to retrospectively evaluate the radiographic marginal bone loss (MBL) and survival rate of NDIs from three different systems after 2 years of functional loading.

**Methods:**

In this retrospective study, a total of 109 NDIs from three brands (30 Straumann^®^, 38 Thommen, and 41 Osstem) in 78 patients were evaluated. Patient records from 2013 to 2023 were screened. Mesial and distal MBL were measured on periapical radiographs at implant loading and at 3, 6, 12, and 24-month follow-ups. MBL was compared based on implant system, gender, and implant location (anterior/posterior, maxilla/mandible).

**Results:**

The overall 2-year implant survival rate was 98.17%. Two implants failed within the first 6 months. The mean MBL at 24 months was 1.67 ± 0.66 mm. Straumann^®^ implants (made of TiZr alloy) showed significantly less MBL than Osstem implants (made of Grade IV titanium) at multiple time points (P < 0.05). Female patients exhibited significantly higher mesial MBL than male patients at 6 and 12 months (P < 0.05). Implants in the posterior region showed greater mesial MBL than those in the anterior region at 3 months (P < 0.05).

**Conclusion:**

Within the limitations of this study, NDIs demonstrated a high 2-year survival rate, supporting their use in anatomically restricted sites. The implant system, particularly the material and design, significantly influenced MBL. The TiZr alloy implant system was associated with the least bone loss. Patient- and site-specific factors, such as gender and posterior location, were also associated with greater MBL in the early healing phase, highlighting the multifactorial nature of peri-implant bone preservation.

## Introduction

The dental implant has become a favored treatment modality for the restoration of partial and complete edentulism (Messias et al., 2021; Bedrossian and Bedrossian, 2019). High success rates (>90%) and excellent predictability of dental implant treatment have been demonstrated in numerous clinical studies and a multiplicity of indications (Moraschini et al., 2015; Smith Nobrega et al., 2016; Pathak et al., 2023). However, the application of standard diameter implants is limited by factors such as a narrow alveolar ridge, insufficient distance between adjacent natural tooth roots, and limited mesiodistal space in the edentulous area (Buser et al., 2012). As a result, additional treatment like bone augmentation is often required to increase insufficient bone volume. However, these surgical procedures are time-consuming and costly, and require advanced surgical experience.

Therefore, alternative concepts such as narrow-diameter implants (NDIs) are becoming of increasing clinical and scientific interest. Narrow-diameter implants (NDIs) are generally considered to have a diameter ≤3.5 mm and are claimed to be a reasonable alternative to bone augmentation procedures, especially for limited interdental space (Schi et al., 2018; Froum et al., 2007). Moreover, it has been reported that NDIs have competitive treatment outcomes compared to regular-diameter implants (RDIs) in terms of implant survival rate and marginal bone loss (MBL) (Park et al., 2023; Kim et al., 2023). However, compared with RDIs, the smaller implant diameter reduces the bone-to-implant contact area and mechanical strength, which could potentially compromise osseointegration and long-term stability (Ormianer et al., 2012). Therefore, clinicians often monitor the MBL around NDIs via radiography to assess treatment success. This study aimed to further evaluate the clinical performance of NDIs from three different systems by measuring and analyzing radiographic MBL over a 2-year functional loading period.

## Materials and methods

### Patients

In this retrospective study, the patient cohort was retrospectively identified from records at the Affiliated Stomatological Hospital of Dalian Medical University for treatments performed between 2013 and 2021, and at the Wuhan No.1 Hospital for treatments performed between 2021 and 2023. The clinical information and digital radiographs (periapical) were collected. Patient records were screened for eligibility based on the inclusion and exclusion criteria. All consecutive patients who met these criteria and had a complete radiographic record up to the 24-month follow-up visit were included in the final analysis. No patients were lost to follow-up within the 24-month observation period after loading. A formal sample size calculation was not performed due to the retrospective nature of the study; the sample size was determined by the number of all available patient records that met the inclusion criteria within the specified timeframe. Information on smoking habits and systemic conditions like diabetes was recorded from patient charts where available. However, due to inconsistent documentation and the retrospective nature of the study, a formal analysis of these potential confounders could not be performed.

Patients who had received NDIs, attended regular follow-up visits, and had signed informed consent were included. Inclusion criteria were: (1) age >18 years; (2) buccolingual or mesiodistal bone width ≤5.50 mm in the edentulous area; (3) useable bone height >10 mm; (4) adequate interocclusal space (>5 mm); (5) healthy periodontal mucosa with sufficient attached gingiva; (6) general health suitable for implant surgery.

Exclusion criteria were: (1) previous bone augmentation surgery at the implant site; (2) tooth loss due to malignancy; (3) acute oral inflammation; (4) history of alcohol abuse or psychiatric illness; (5) systemic conditions contraindicating surgery; (6) unmanaged systemic bone diseases such as severe osteoporosis; (7) local soft or hard tissue lesions.

Finally, a total of 78 patients (47 males, 31 females) were enrolled, with an age range of 25–81 years (mean age: 55 years).

### Implants

A total of 109 implants were analyzed. These included three different NDI systems: 30 Straumann^®^ Bone Level Tapered (BLT) implants (Straumann Holding AG, Basel, Switzerland) with a diameter of 3.3 mm, made of TiZr alloy (Roxolid^®^) with an SLActive^®^ surface and a Morse taper internal connection; 38 Thommen SPI^®^ ELEMENT MC implants (Thommen Medical AG, Grenchen, Switzerland) with a diameter of 3.5 mm, made of Grade IV titanium with an ApliFlex^®^ surface and an internal hexagonal connection; and 41 Osstem TSIII SA implants (Osstem Implant Co., Ltd., Seoul, Korea) with a diameter of 3.5 mm, made of Grade IV titanium with an SA (sandblasted with alumina and acid-etched) surface and an internal conical hex connection. These three systems were chosen for evaluation as they were the most frequently used NDIs at the participating centers during the study period and represent distinct differences in material, surface technology, and connection design, providing a relevant basis for clinical comparison. The distribution of the 109 implants is as follows: 43 in females and 66 in males; 76 in the anterior region and 33 in the posterior region; 69 in the maxilla and 40 in the mandible. A detailed distribution of implants by system, location, and jaw is presented in Supplementary Table S1.

### Surgical and prosthetic procedure

All patients underwent professional oral hygiene procedures before surgery. Local infiltration anesthesia was administered using 4% articaine. A crestal incision, with or without releasing incisions as needed, was made to expose the alveolar ridge. NDIs were placed according to the manufacturer’s recommended protocol, achieving a final insertion torque of ≥30 N cm. Periapical radiographs were taken immediately post-surgery. Post-operative care included oral anti-inflammatory medication, analgesics, and instructions for cold pack application. Patients were instructed to use a 0.12% chlorhexidine mouthwash daily until suture removal. For two-stage protocols, second-stage surgery was performed 3 months after placement. Three weeks later, prosthetic rehabilitation was completed. The prosthetic procedure was performed by a restorative dentist/prosthodontist. Prosthetic restorations included cement- or screw-retained single crowns or fixed partial dentures made of porcelain-fused-to-metal (PFM) or zirconia. All implant surgeries were performed by experienced clinicians (with >5 years of implant surgical experience). Follow-up radiographic examinations were conducted at 3, 6, 12, and 24 months after final prosthesis delivery.

### Radiographic examination and measurements

**FIGURE 1:**
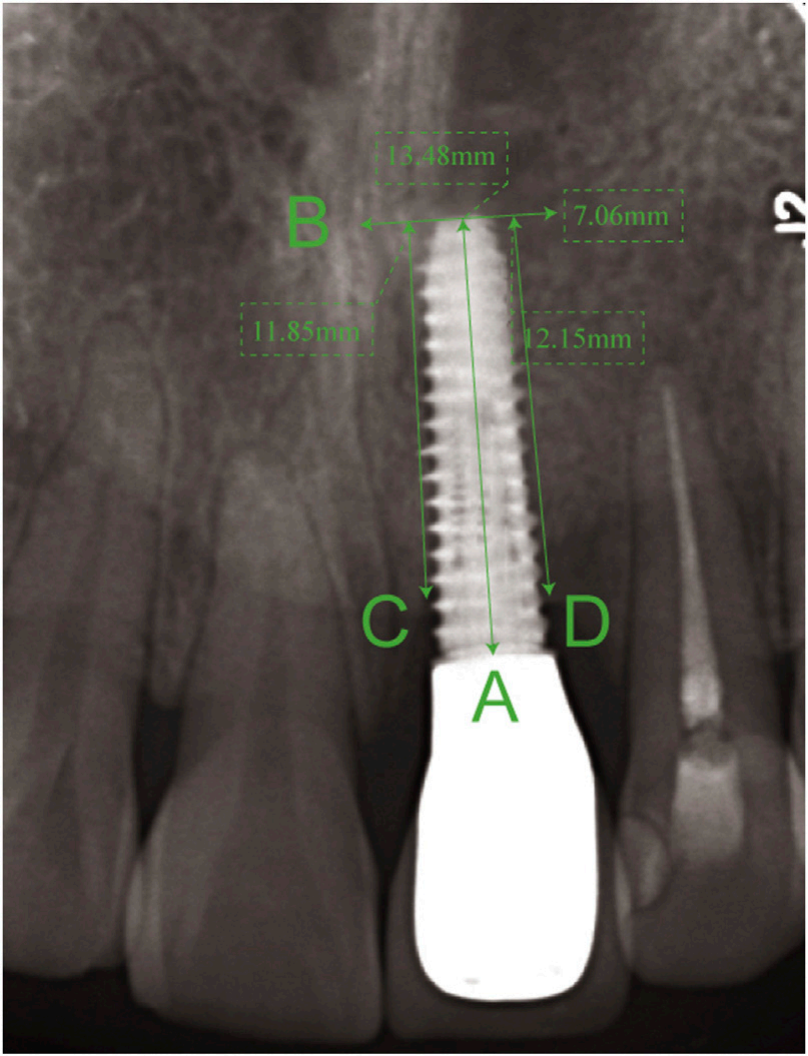
Schematic diagram for the measurement of marginal bone loss (MBL). The known implant length (A) is used for calibration. The MBL is calculated as the vertical distance from the implant shoulder (B) to the first bone-to-implant contact on the mesial (C) and distal (D) aspects, measured parallel to the implant’s long axis.

All patients received standardized periapical X-ray examinations at baseline (implant placement), loading (prosthesis delivery), and at the 3-, 6-, 12-, and 24-month follow-up visits. Radiographs were taken using a standardized parallel technique with a beam-aiming device and digital sensor holders (Rinn XCP, Dentsply Sirona, York, PA, United States) to ensure reproducibility. MBL was defined as the vertical distance from the implant shoulder to the most coronal point of bone-to-implant contact, measured parallel to the long axis of the implant on both mesial and distal aspects using calibrated imaging software (Sidexis XG, Dentsply Sirona). Measurements were calibrated using the known length of the implant as a reference (Figure 1). MBL at each follow-up was calculated as the difference between the bone level at that time point and the bone level at the time of loading. To ensure measurement reliability, one calibrated examiner (X.F.) performed all measurements. To assess intra-examiner reliability, 20 randomly selected radiographs were re-measured by the same examiner 2 weeks after the initial assessment. To assess inter-examiner reliability, a second calibrated examiner (X.C.) independently measured the same 20 radiographs. The Intraclass Correlation Coefficient (ICC) was calculated. The intra-examiner and inter-examiner reliability were excellent (ICC = 0.98 and 0.96, respectively), indicating a high degree of measurement consistency.

### Statistical analysis

All statistical analyses were conducted using SPSS 26.0 statistical software (IBM Corp., Armonk, NY, United States). The normality of data distribution was assessed using the Shapiro-Wilk test, and the homogeneity of variances was checked with Levene’s test. Measurement data were expressed as mean ± standard deviation (SD), median, and range. For comparisons between two groups (e.g., gender, jaw location), the independent samples t-test was used if data were normally distributed and variances were equal; otherwise, the Mann-Whitney U test was applied. For comparisons among the three implant systems, a one-way analysis of variance (ANOVA) was performed, followed by a Bonferroni post-hoc test for pairwise comparisons to correct for multiple testing. The chi-squared test was used to compare implant survival rates. A P-value <0.05 was considered statistically significant.

## Results

### Baseline information

The study included 47 males (mean age: 53 ± 14 years) and 31 females (mean age: 56 ± 17 years). There was no statistically significant difference in age between genders (P > 0.05).

### Oral implant survival

At the 6-month post-loading follow-up, two implants had failed. The first failure involved a Thommen implant in the maxillary right central incisor site of a 58-year-old female patient, who was a known heavy smoker (approximately 20 cigarettes per day). The failure was characterized by mobility and progressive bone loss, noted at the 6-month visit. The second failure involved an Osstem implant in the mandibular right incisor site of a 45-year-old male with a history of poor oral hygiene and suspected uncontrolled bruxism. The overall 2-year implant survival rate was 98.17% (107/109). There was no statistically significant difference in survival rates among the three implant systems (P > 0.05, Chi-squared test).

### Marginal bone loss in different systems

**FIGURE 2:**
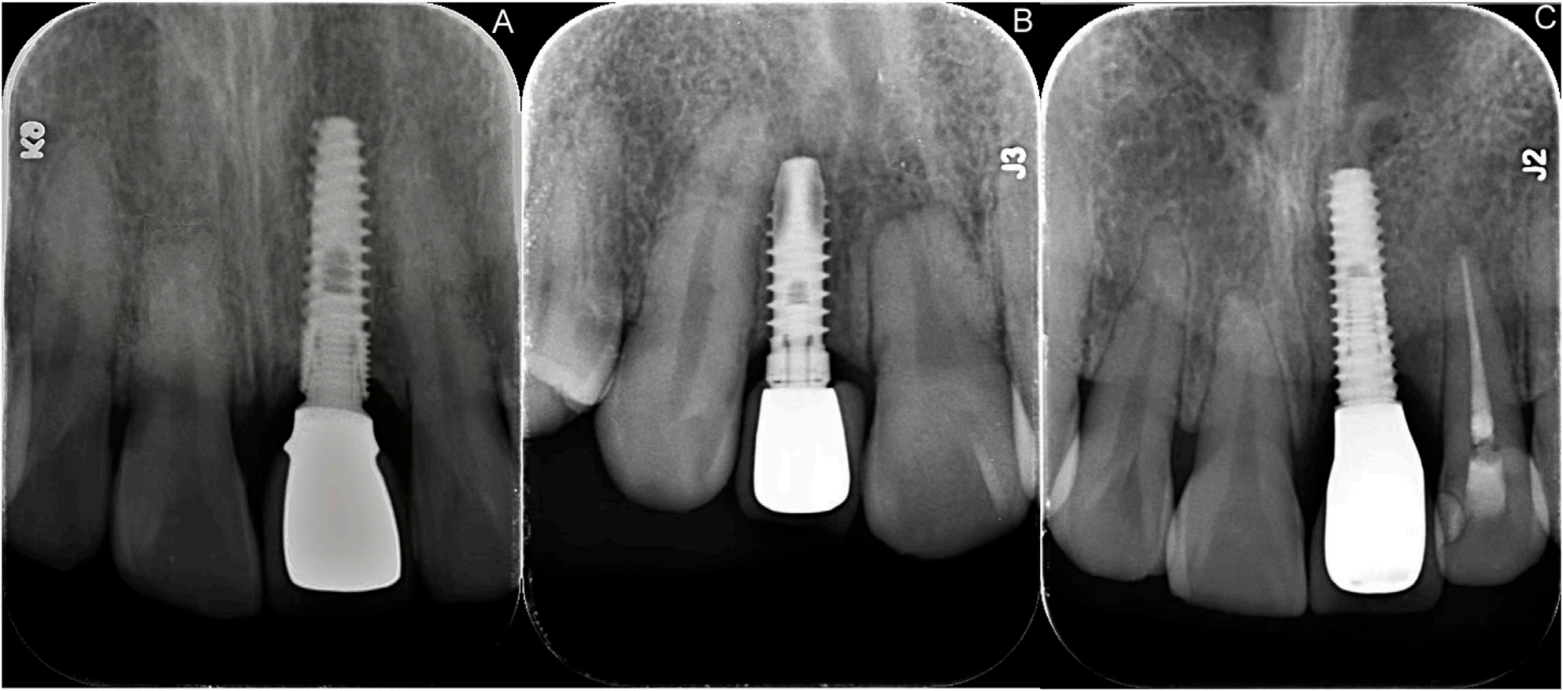
Representative periapical radiographs showing MBL at the 24-month follow-up for the three implant systems. **(A)** Straumann^®^ BLT implant. **(B)** Thommen SPI^®^ ELEMENT implant. **(C)** Osstem TSIII SA implant.

The mean MBL for all implants was 1.49 ± 0.83 mm at 1 year and 1.67 ± 0.66 mm at 2 years. Throughout the observation period, the MBL of Osstem implants was significantly higher than that of Straumann implants (P < 0.05). At 3 months, the distal MBL of Thommen implants was also significantly higher than that of Straumann implants (P < 0.05). No other significant differences were found among the three implant systems at other time points. These results are detailed in Table 1 and illustrated with representative examples in Figure 2.

**TABLE 1:** Marginal bone loss changes in three implant systems at different periods (mean ± SD, mm).

Group	Area	After 3 months	After 6 months	After 12 months	After 24 months
Osstem	The mesial MBL	1.07 ± 0.51*^a^	1.44 ± 0.71*^a^	1.46 ± 0.67	1.88 ± 0.59
	The distal MBL	0.91 ± 0.43*^c^	1.49 ± 0.59	1.52 ± 0.71	2.11 ± 0.62
Thommen	The mesial MBL	0.56 ± 0.34*^a^ ^,^ ^b^	0.97 ± 0.67*^a^ ^,^ ^b^	1.20 ± 0.49	1.23 ± 0.17
	The distal MBL	0.84 ± 0.37*^b^	1.20 ± 0.64*^b^	1.55 ± 0.89	1.56 ± 0.12
Straumann	The mesial MBL	0.64 ± 0.39*^b^	0.87 ± 0.42*^b^	1.06 ± 0.37	1.29 ± 0.56
	The distal MBL	0.46 ± 0.36*^b^ ^,^ ^c^	0.88 ± 0.13*^b^	1.14 ± 0.10	1.46 ± 0.52

Note: *P < 0.05 after Bonferroni correction.

^a^ Comparison between Osstem and Thommen implants.

^b^ Comparison between Thommen and Straumann implants.

^c^ Comparison between Osstem and Straumann implants.

### Marginal bone loss in different genders and areas

At the 6- and 12-month follow-ups, the mesial MBL in female patients was significantly higher than in male patients (P < 0.05, Table 2). At 3 months, the mesial MBL was significantly greater in the posterior region compared to the anterior region (P < 0.05, Table 3). There were no other statistically significant differences for other variables (P > 0.05). A detailed breakdown of the 24-month MBL data, including median and range, for different subgroups is presented in Table 4.

**TABLE 2:** Marginal bone loss changes between genders (mean ± SD, mm).

Gender	Area	After 3 months	After 6 months	After 12 months	After 24 months
Male	The mesial MBL	0.89 ± 0.47	0.97 ± 0.77	1.01 ± 0.39	1.50 ± 0.59
	The distal MBL	0.73 ± 0.38	1.21 ± 0.56	1.37 ± 0.81	1.66 ± 0.97
Female	The mesial MBL	0.87 ± 0.49	1.67 ± 0.66	1.88 ± 0.64	1.75 ± 0.44
	The distal MBL	0.86 ± 0.40	1.44 ± 0.70	1.46 ± 0.65	1.96 ± 0.43
p-value	The mesial MBL	0.861	<0.001**	<0.001**	0.056
	The distal MBL	0.164	0.122	0.619	0.127

Note: **P < 0.001.

**TABLE 3:** Marginal bone loss changes in different implant regions (mean ± SD, mm).

Group	Area	After 3 months	After 6 months	After 12 months	After 24 months
Posterior	The mesial MBL	0.50 ± 0.46	0.86 ± 0.40	1.23 ± 0.35	1.60 ± 0.46
	The distal MBL	0.87 ± 0.49	1.01 ± 0.32	1.38 ± 0.36	1.77 ± 0.53
Anterior	The mesial MBL	0.86 ± 0.54	0.81 ± 0.46	1.46 ± 0.90	1.66 ± 0.58
	The distal MBL	0.97 ± 0.45	1.25 ± 0.64	1.27 ± 0.69	1.57 ± 0.70
p-value	The mesial MBL	0.005*	0.638	0.207	0.285
	The distal MBL	0.374	0.073	0.444	0.201

Note: *P < 0.05.

**TABLE 4:** Descriptive statistics of marginal bone loss (MBL) at 24-month follow-up for different subgroups (mm).

Subgroup	Category	N (Implants)	Mean ± SD	Median	Range (Min–Max)
Implant System	Straumann	30	1.38 ± 0.54	1.35	0.50–2.45
	Thommen	38	1.40 ± 0.15	1.40	1.10–1.70
	Osstem	41	1.99 ± 0.61	1.95	0.90–3.10
Gender	Male	66	1.58 ± 0.78	1.55	0.50–2.90
	Female	43	1.85 ± 0.44	1.80	0.95–3.10
Location	Anterior	76	1.62 ± 0.64	1.60	0.50–3.10
	Posterior	33	1.68 ± 0.50	1.70	0.80–2.60

## Discussion

Bone resorption at the implant margin is a critical factor affecting long-term implant stability. According to the historical success criteria proposed by Albrektsson et al. Albrektsson et al. (1986), MBL should be less than 1.5 mm in the first year of loading, with subsequent annual resorption of less than 0.2 mm. In this study, the mean MBL was 1.49 ± 0.83 mm at the first year and 1.67 ± 0.66 mm at the second year, which approached the upper limit of this historical benchmark. While these values are near the threshold defined by Albrektsson et al. (1986), it is important to note that more recent literature suggests stricter criteria for success. For instance, studies by Galindo-Moreno et al. (2015) and a consensus report by Wang et al. (2025) advocate for MBL ideally not exceeding 0.5 mm–1.0 mm during the first year of function to be considered a truly successful outcome in contemporary implantology. Therefore, while our findings may be considered acceptable by older standards, the observed MBL could be viewed as significant by current, more stringent benchmarks, highlighting an area for ongoing improvement and careful long-term monitoring.

There are obvious differences among implant systems in terms of morphology, material, surface treatment, and connection design (Rahimi et al., 2022). In this study, the Straumann TiZr implants showed the least MBL over the observation period. The Straumann BLT implants used here feature a TiZr alloy (Roxolid^®^), known for its high mechanical strength, and a Morse taper internal connection, which is designed to provide a tight seal and minimize micromovement. These features may contribute to enhanced biomechanical stability and a reduced risk of bacterial leakage, potentially leading to less MBL (Bernhard and e t al., 2009). Our findings on the performance of the TiZr alloy implant are consistent with a meta-analysis by Altuna et al. (2016), which also reported low MBL and high survival rates for these implants. In contrast, the Osstem TSIII SA implants, made of Grade IV titanium with an internal conical hex connection, showed the highest MBL. The clinical significance of the statistically different MBL between the implant systems, particularly between Straumann and Osstem, warrants consideration. While a mean difference of approximately 0.6 mm at 2 years may not immediately compromise implant survival, it could be an early indicator of differing long-term susceptibility to peri-implant complications. The superior performance of the TiZr alloy implant with a Morse taper connection in this study may suggest better biomechanical stability and a more effective seal against bacterial leakage, factors known to influence peri-implant bone preservation. Continuous monitoring beyond 2 years is essential to determine if these early patterns in MBL translate to divergent long-term survival and success rates.

With societal aging, the prevalence of osteoporosis increases, particularly among postmenopausal women. The decline in estrogen levels leads to an imbalance in bone metabolism, favoring bone resorption (Cheng et al., 2022). While our study did not directly measure hormonal status or diagnose osteoporosis, the finding of higher MBL in female patients (average age 56 years) aligns with the extensive body of literature suggesting that postmenopausal estrogen deficiency can negatively impact bone metabolism and peri-implant health (Hernández-Vigueras et al., 2016). Thus, the observed gender difference in our cohort may reflect underlying biological factors associated with age and menopause. However, this interpretation should be viewed with caution given the retrospective nature of our study and the lack of hormonal data. Meta-analyses and randomized controlled trials have found that systemic hormone therapy in postmenopausal women can improve bone mineral density, confirming that managing systemic bone health is an effective way to prevent osteoporosis and related fractures (Papadakis et al., 2016; Lindsay et al., 1997; Farquhar et al., 2009). This suggests that for at-risk patient populations, a collaborative approach with a physician regarding systemic bone health may be beneficial for long-term implant success.

NDIs were originally designed for areas with limited mesiodistal space, such as maxillary lateral incisors and mandibular incisors (Schi et al., 2018; Cruz et al., 2021). Their use in the posterior region, particularly for molar replacement, has been debated due to higher occlusal forces. The high survival rate (98.17%) observed in our study is strongly supported by recent literature. For instance, a large systematic review by Valente et al. (2022), analyzing over 1,200 NDIs, reported a comparable cumulative survival rate of 97.80%, confirming the high predictability of this treatment modality in atrophic ridges. In this study, NDIs placed in the posterior (bicuspid) region had a 100% survival rate, though they exhibited greater MBL initially than anterior implants. Other studies support the use of NDIs in the bicuspid region as a reliable alternative to bone augmentation (Papadimitriou et al., 2015). However, biomechanical studies show that stress in the surrounding alveolar bone increases as implant diameter decreases, so caution is still advised for molar replacement (Ormianer et al., 2012). Long-term clinical observation is needed to establish definitive guidelines.

This study has several limitations inherent to its retrospective design. Firstly, the design is susceptible to selection bias, and some patient data, such as smoking status and precise oral hygiene metrics, were inconsistently recorded, precluding robust subgroup analysis of these important confounders. Secondly, a formal sample size calculation was not performed, and the sample size was based on convenience, which may limit the statistical power to detect smaller differences between groups. Thirdly, the 2-year follow-up period provides valuable short-to medium-term data, but longer-term observation is necessary to ascertain the ultimate longevity and MBL patterns of these implants. Finally, the multicenter nature of data collection over a long period may introduce variability in surgical and prosthetic protocols, despite efforts to include cases treated by experienced clinicians. Future prospective, randomized controlled trials with larger sample sizes and standardized protocols are warranted to corroborate these findings.

## Conclusion

Within the limitations of this retrospective study, NDIs from all three systems demonstrated a high 2-year survival rate, supporting their clinical utility as a viable treatment option in anatomically restricted sites. The choice of implant system, particularly its material and design, was found to be a significant factor influencing peri-implant MBL, with the TiZr alloy implant system (Straumann^®^) exhibiting significantly less bone loss than the Grade IV titanium systems. Furthermore, patient- and site-specific factors, such as female gender and posterior implant location, were associated with greater MBL in the initial healing stages. These findings underscore that NDI treatment outcomes are multifactorial and highlight the importance of careful implant selection and patient evaluation for optimizing long-term success.

## Data Availability

The original contributions presented in the study are included in the article/Supplementary Material, further inquiries can be directed to the corresponding author.

## Abbreviations

ANOVA, Analysis of Variance; HT, Hormonal Therapy; ICC, Intraclass Correlation Coefficient; MBL, Marginal Bone Loss; NDIs, Narrow-Diameter Implants; PFM, Porcelain-Fused-to-Metal; RDIs, Regular-Diameter Implants; SD, Standard Deviation; TiZr, Titanium-Zirconium Alloy.
