# Supplementary material for: Radiographic evaluation of narrow-diameter implants from different systems for two years
Source: Front Bioeng Biotechnol. 2025 Sep 1;13:1631745. doi: 10.3389/fbioe.2025.1631745 (PMC12433985; doi:10.3389/fbioe.2025.1631745)
Supplement: Supplementary file 1 [file Table1.docx]

**Supplementary Table 1. Distribution of Implants by System, Location, and Jaw. (n)**

| **Implant System** | **Region** | **Jaw** | **Number of Implants** |
| --- | --- | --- | --- |
| Straumann (Total N=30) | Anterior (n=20) | Upper | 13 |
|  |  | Lower | 7 |
|  | Posterior (n=10) | Upper | 5 |
|  |  | Lower | 5 |
| Thommen (Total N=38) | Anterior (n=28) | Upper | 20 |
|  |  | Lower | 8 |
|  | Posterior (n=10) | Upper | 6 |
|  |  | Lower | 4 |
| Osstem (Total N=41) | Anterior (n=28) | Upper | 18 |
|  |  | Lower | 10 |
|  | Posterior (n=13) | Upper | 7 |
|  |  | Lower | 6 |
| **Total Anterior Implants** |  | | **76** |
| **Total Posterior Implants** |  | | **33** |
| **Total Upper Jaw Implants** |  | | **69** |
| **Total Lower Jaw Implants** |  | | **40** |
| **Grand Total Implants** |  | | **109** |
